# Assessing gait variability concurrently with dynamic visual acuity on a treadmill in people with bilateral vestibulopathy

**DOI:** 10.1177/09574271251313806

**Published:** 2025-01-10

**Authors:** Christopher McCrum, Meichan Zhu, Paul Willems, Ivar Visser, Melina Kastrinou, Raymond van de Berg, Kenneth Meijer, Ann Hallemans, Nolan Herssens

**Affiliations:** 1 Department of Nutrition and Movement Sciences, NUTRIM School of Nutrition and Translational Research in Metabolism, 199236 Maastricht University Medical Centre+, Maastricht, The Netherlands; 2 Division of Balance Disorders, Department of Otorhinolaryngology and Head and Neck Surgery, Faculty of Health Medicine and Life Sciences, School for Mental Health and Neuroscience, 199236 Maastricht University Medical Centre+, Maastricht, The Netherlands; 3 Department of Rehabilitation Sciences and Physiotherapy/MOVANT, Faculty of Medicine and Health Sciences, 26660 University of Antwerp, Wilrijk, Belgium; 4 Space Medicine Team (HRE-OM), European Astronaut Centre, European Space Agency, Cologne, Germany

**Keywords:** Balance, gait analysis, vestibular system, walking

## Abstract

**Background:**

Gait variability is increased in people with bilateral vestibulopathy (BVP). Since dedicated gait analysis can be resource-intensive, concurrent assessment with another vestibular function test, dynamic visual acuity (DVA), is worth consideration.

**Objective:**

To assess comparability of results from a combined gait and DVA assessment with results from a previous dedicated gait analysis.

**Methods:**

15 participants (4 women) with BVP were analysed. The DVA test assessed visual acuity during stance and during treadmill walking at 2, 4 and 6 km/h. An 8-camera motion capture system measured spatiotemporal gait parameters (step length, step time, step width and double support time; means and coefficients of variation [CoV]). The walking speed effect was assessed by mixed-effects models, and results were visually compared to previous results.

**Results:**

Walking speed affected the means of step length, step time and double support time (*p* < .0001) but not step width (*p* = .373) and significantly affected the CoV of all parameters (*p* < .01). These values, as well as speed-related changes, were comparable between contexts.

**Conclusions:**

Concurrent DVA and gait assessment seems promising as an assessment method in people with BVP. Test-retest reliability, clinically feasible motion capture solutions and sensitivity to change following interventions should be further investigated.

## Introduction

Bilateral vestibulopathy (BVP), categorized by significant bilateral impairment or absence of vestibular function,^1,2^ leads to various physical, cognitive and emotional complaints, often contributing to a diminished quality of life.^3^ Among these complaints, reduced balance performance,^4,5^ an increased risk of falls,^6–9^ and increased variability of spatiotemporal gait parameters^8,10,11^ can severely affect patients’ mobility. Clinical tests of vestibular function do not correspond well to increased risk of falls^8,12^ or to spatiotemporal gait variability parameters in people with BVP,^10^ indicating that tests of gait and balance are an important addition to vestibular function testing. However, gait analysis can be costly, time consuming and require specific expertise and equipment. Integration of gait analysis with other tests used to evaluate people with BVP may help address some of these issues.

One test of vestibular function in BVP that may be well suited to a combination with gait analysis is dynamic visual acuity (DVA) testing.^13–16^ DVA refers to the extent to which the visual system can maintain clear and detailed visual perception of objects during head movements^17^ and can be tested by comparing visual acuity during a static condition (i.e., no/little head movement) to visual acuity during a dynamic condition such as walking (i.e., with head movement). This is commonly conducted in research settings in people with BVP over multiple different walking speeds (e.g., 2, 4 and 6 km/h) on a treadmill, meaning that potential walking speed effects on gait parameters of interest can also be investigated by a combined testing approach. However, due to differences in task duration (and thereby number of steps measured) and requirements (e.g., concurrent walking and DVA assessment may function as a cognitive dual-task and could lead to deteriorations in gait^18,19^), it is currently unclear if mean and variability values of spatiotemporal gait parameters assessed during DVA testing will closely replicate previous values found in more dedicated gait analysis setups and protocols. In our previous study, we found that step length, step time and double support time variability could distinguish between the healthy participants and participants with BVP, and between people with BVP with different locomotor capacities.^10^

In a previous study, people with bilateral vestibulopathy completed a dynamic visual acuity test on a treadmill.^12^ While not analysed or reported in the previous publication, motion capture data was recorded during most of these trials. In this study, we analysed the spatiotemporal gait parameters of these participants during the different speeds of the DVA test. In order to assess if combined gait and DVA analysis could have potential as an efficient assessment method for people with BVP, we aimed to determine (1) if we could replicate the statistically significant effects of walking speed on the spatiotemporal parameters and their variability found previously^10^ and (2) if the means and variability of the parameters determined in this way were similar to those reported in a dedicated gait analysis protocol^10^ in a different cohort of people with BVP.

## Methods

### Participants

The data recorded during the DVA testing analysed in this study were collected previously as part of a study approved by the Ethics Committee of the University of Antwerp/Antwerp University Hospital (B300201629697). All participants gave written informed consent at the time of study inclusion. All participants included in the previous study met the diagnostic criteria of bilateral vestibulopathy as proposed by the Barany Society^1,2^: (1) a horizontal angular vestibulo-ocular reflex (VOR) gain <0.6, as measured by the video Head Impulse Test (vHIT) and/or (2) a reduced caloric response (i.e., sum of bithermal, 30 and 44°C, maximum peak slow-phase velocity on each side <6°/s) and/or (3) a reduced horizontal angular VOR gain ≤0.1 upon sinusoidal stimulation on a rotatory chair. The DVA results were analysed and discussed in a previous publication.^12^ From the convenience sample of 27 BVP patients included in the previous study,^12^ ten had missing marker data or large gaps in marker data and could not be processed. Two did not have motion capture recordings. This left viable motion capture data of 15 participants (11 men and 4 women). These 15 participants had mean (SD) age of 54.5(10.5) years, height of 172.6(9.5)cm, and weight of 75.4(17.3)kg. Given the secondary nature of the analysis, no formal sample size calculation was performed *a priori*.

Since the DVA assessment duration is dependent on the time taken to determine the visual acuity based on the line at which more than two optotypes were not read correctly, the number of steps available for analysis per speed was not known *a priori*. Some trials also suffered from data quality issues (the motion capture part of the original data collection was not the primary purpose of the study). After data processing and cleaning, the number of steps included in the analyses varied across the participants and these are presented in Table 1 for additional context. Note that some trials are below the number of steps typically recommended for gait variability analysis,^20,21^ but due to our aim relating to gait analysis conducted during standard DVA assessment, we did not exclude these trials from the analysis.

**Table 1.** Number of steps analysed for each participant at each walking speed.

	Walking speed (km/h)			Reasons for missing or reduced data^a^
Participant Code	**2**	**4**	**6**	
BV01	31	118		Poor marker data quality for 2 km/h. 6 km/h was too fast.
BV02	69	98	115	
BV03	16	14		2 km/h and 4 km/h could not be maintained for long. 6 km/h was too fast.
BV04	6	102	116	Poor marker data quality for 2 km/h.
BV05	96	117	129	
BV06	33	7		2 km/h and 4 km/h could not be maintained for long and had poor marker data quality. 6 km/h was too fast.
BV07	83	104	123	
BV08	60	94	119	
BV09	70	24	118	Poor marker data quality for 4 km/h.
BV10	62	69	146	
BV11	15	52	53	Poor marker data quality for 2 km/h.
BV12	41	75	124	Poor marker data quality for 2 km/h.
BV13	74	104	107	
BV14	79	13	118	Poor marker data quality for 4 km/h.
BV15	71	100	119	

^a^It was noted that the marker data quality in terms of amount and size of gaps was suboptimal during all 2 km/h trials, mostly due to the lack of arm swing at this speed (affecting trochanter marker visibility) combined with the treadmill frame occluding trochanter and foot markers for longer time periods than at the faster speeds. This caused issues in our semi-automatic marker labelling and gap filling routines. Since we wanted to perform the data processing in the same manner as our previous study, we did not perform any additional, manual processing steps and rather excluded these portions of the data. Only 2 km/h trials for which this had a severe effect on the number of steps analysed are detailed in the table.

### Dynamic visual acuity testing

The DVA test was prepared as described in Verbecque et al.^22^ Full technical details of the DVA assessment and setup can also found in Herssens et al.^12^ Here, we detail only the parts of the assessment relevant for the gait assessment. Revised 2000 Series EDTRS charts with Sloan letters (CDHKNORSVZ) were used with rows of five randomly chosen letters. LogMAR scale and notation were used. Each participant read the optotypes aloud to determine the visual acuity and started reading at the 0.4 logMAR line. Whenever the participant was unable to read all optotypes correctly, they were instructed to read the line above (0.5 logMAR), which was repeated until all optotypes on the same line were read correctly. Otherwise, the participant had to read lines with decreasing optotype size until >2 optotypes were missed on a single line. The assessment began with determining static visual acuity while participants stood still on the treadmill keeping their head still. Following this, DVA was assessed while walking on the treadmill at 2, 4 and 6 km/h. The speed conditions were non-randomized, and the participants were allowed a familiarization period of 1 minute for each walking speed. As a result of the nature of the DVA assessment, there was not a standardized number of minutes or steps walking. The test procedure was stopped if the participant or researcher felt that they could not complete the walking speed trial safely. The participants wore a safety harness attached to an overhead frame to avoid falls while standing and walking on the treadmill. Participants were strongly encouraged not to use the treadmill handrails and if handrail use was deemed to be too much by the researcher, indicating that the participant could not walk safely without support, then the testing was stopped for that walking speed.

### Data processing

An 8-camera motion capture system (Vicon T10, 100 Hz., Vicon Motion Systems Ltd., Oxford, UK) was used to track reflective markers placed on anatomical landmarks of the participants’ body, corresponding to the left and right hallux, lateral malleolus, major trochanter, the sacrum and C7. The motion capture data was processed in as similar a manner as possible to our previous dedicated gait analysis,^10^ the major difference being a lack of force plate in the treadmill. Specifically, marker data were filtered using a low-pass second-order Butterworth filter (zero-phase) with a 12 Hz cut-off frequency. Heel strike and toe off were determined using the method of Zeni et al.^23^ The spatiotemporal gait parameters of interest were step length (anteroposterior distance between the hallux markers at foot touchdown), step time (time from touchdown of one foot to touchdown of the next foot), step width (mediolateral distance between the hallux markers at foot touchdown) and double support time (time spent with both feet on the ground). Means and coefficients of variation (CoV) were determined for each speed for each participant.

### Statistics

**Figure 1.**
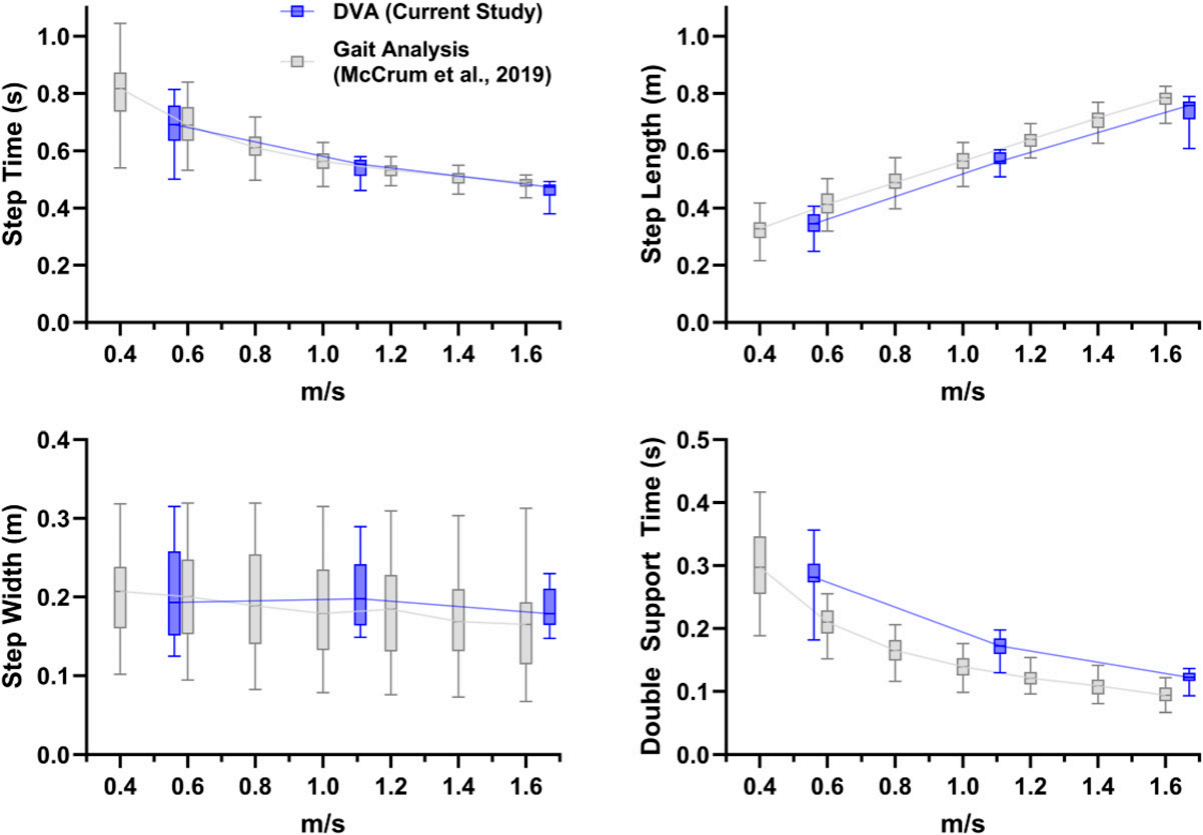
Boxplots (median, interquartile range and 5^th^ to 95^th^ percentiles) of the mean values of the spatiotemporal gait variables for the current data collected during the DVA assessment (dark blue; 2 km/h, 4 km/h and 6 km/h [0.56 m/s, 1.11 m/s and 1.67 m/s, respectively]) and for data collected during a dedicated gait analysis from our previous study (light grey^10^).

**Figure 2.**
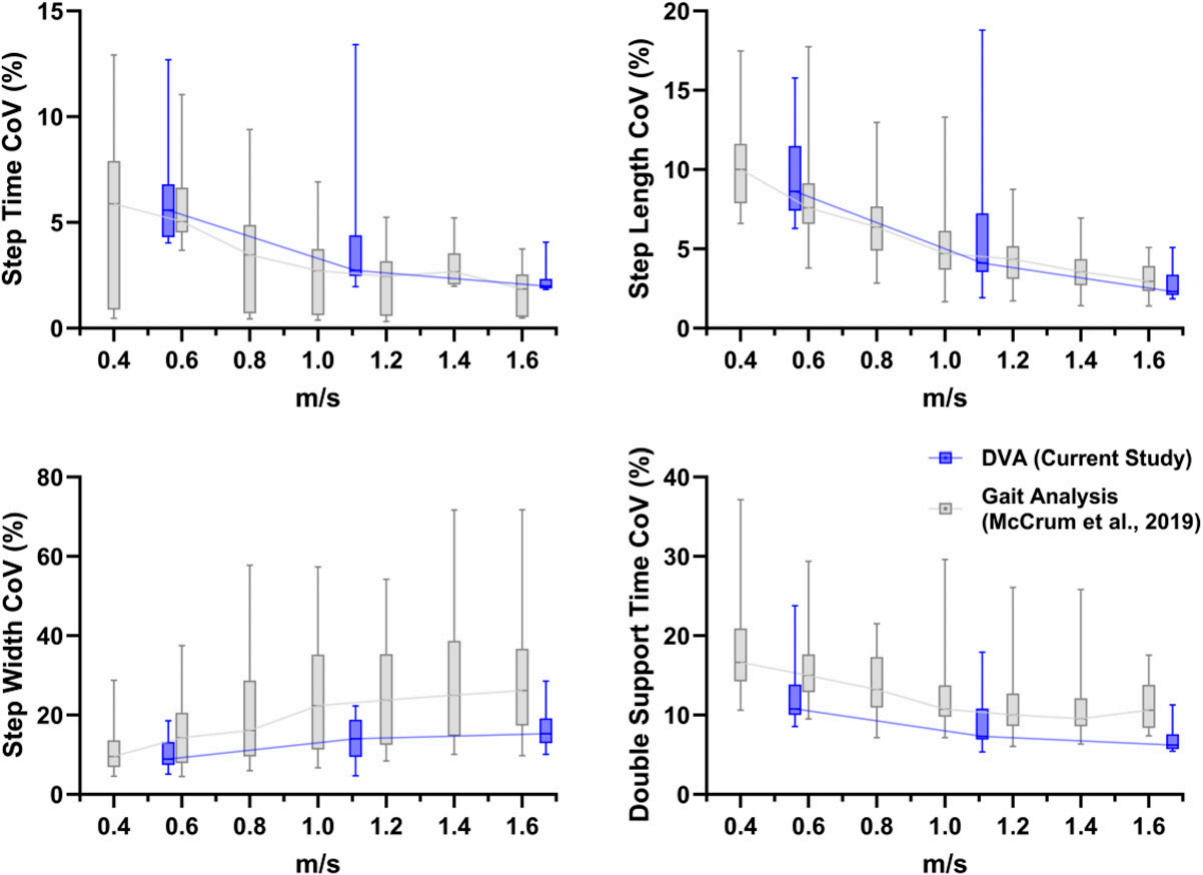
Boxplots (median, interquartile range and 5^th^ to 95^th^ percentiles) of the coefficient of variation values of the spatiotemporal gait variables for the current data collected during the DVA assessment (dark blue; 2 km/h, 4 km/h and 6 km/h [0.56 m/s, 1.11 m/s and 1.67 m/s, respectively]) and for data collected during a dedicated gait analysis from our previous study (light grey^10^).

To assess the effect of walking speed on the gait parameters, mixed-effects analyses with walking speed as a repeated measures factor with Geisser-Greenhouse sphericity correction were conducted, with Bonferroni-corrected pairwise comparisons (*p* values of the pairwise comparisons are displayed in their corrected form). The threshold for statistical significance was set at *p* = .05. Analyses were conducted using GraphPad Prism version 10.1.1 for Windows (GraphPad Software, Boston, Massachusetts USA, https://www.graphpad.com). Results were also compared visually with those of our previous study^10^ by overlaying the results on the same figure (see Figures 1 and 2). Note that direct statistical comparison of the current and previous study was not done since the walking speeds used differed.

## Results

The mixed-effects analyses revealed significant effects of walking speed on the mean values of step length, step time and double support time (all *p* < .0001 with significant [*p* < .0001] pairwise comparisons between all walking speeds for all three parameters) but not step width (*p* = .373). The mixed-effects analyses revealed significant effects of walking speed on the CoV of all parameters (all *p* < .01 with significant [*p* < .05] pairwise comparisons between all walking speeds for step length, double support time and step width, and between 6 km/h and the other two speeds for step time). Complete results are shown in Tables 2 and 3.

**Table 2.** Results of the mixed-effects models of walking speed on the gait parameters.

	*p*-Value	F (DFn, DFd)	Geisser–Greenhouse’s epsilon
Step time means	<0.0001	F (1.124, 14.05) = 136.1	0.5622
Step length means	<0.0001	F (1.859, 23.23) = 722.3	0.9293
Double support time means	<0.0001	F (1.025, 12.81) = 245.6	0.5123
Step width means	0.3729	F (1.240, 15.50) = 0.9235	0.6199
Step time CoV	0.0005	F (1.693, 21.16) = 12.28	0.8463
Step length CoV	<0.0001	F (1.533, 19.16) = 20.73	0.7666
Double support time CoV	0.0006	F (1.553, 19.41) = 12.71	0.7763
Step width CoV	0.0024	F (1.414, 17.67) = 10.32	0.7069

**Table 3.** Results of the pairwise comparisons between walking speed conditions.

	Bonferroni’s multiple comparisons test	Mean difference	95% CI of difference			Adjusted *p*-value
Step time means (s)	2 km/h vs. 4 km/h	0.152	0.114	to	0.191	<0.0001
	2 km/h vs. 6 km/h	0.229	0.175	to	0.283	<0.0001
	4 km/h vs. 6 km/h	0.077	0.062	to	0.092	<0.0001
Step length means (m)	2 km/h vs. 4 km/h	−0.220	−0.243	to	−0.197	<0.0001
	2 km/h vs. 6 km/h	−0.394	−0.425	to	−0.362	<0.0001
	4 km/h vs. 6 km/h	−0.174	−0.205	to	−0.142	<0.0001
Double support time means (s)	2 km/h vs. 4 km/h	0.118	0.096	to	0.140	<0.0001
	2 km/h vs. 6 km/h	0.168	0.140	to	0.197	<0.0001
	4 km/h vs. 6 km/h	0.050	0.044	to	0.056	<0.0001
Step time CoV (%)	2 km/h vs. 4 km/h	1.914	−0.250	to	4.077	0.0919
	2 km/h vs. 6 km/h	3.702	2.964	to	4.440	<0.0001
	4 km/h vs. 6 km/h	1.788	0.568	to	3.008	0.005
Step length CoV (%)	2 km/h vs. 4 km/h	3.527	0.546	to	6.507	0.0187
	2 km/h vs. 6 km/h	6.664	5.036	to	8.291	<0.0001
	4 km/h vs. 6 km/h	3.137	1.272	to	5.002	0.0018
Double support time CoV (%)	2 km/h vs. 4 km/h	3.994	0.496	to	7.491	0.0233
	2 km/h vs. 6 km/h	5.725	2.982	to	8.468	0.0003
	4 km/h vs. 6 km/h	1.731	0.421	to	3.042	0.01
Step width CoV (%)	2 km/h vs. 4 km/h	−3.950	−7.561	to	−0.339	0.0302
	2 km/h vs. 6 km/h	−6.395	−11.240	to	−1.552	0.0101
	4 km/h vs. 6 km/h	−2.445	−4.744	to	−0.146	0.0363

The results of the current analysis are presented in Figure 1 (mean values) and Figure 2 (CoV values). The current values (blue boxplots) have been overlaid on the results of our previous study,^10^ shown in grey. These show very similar mean and CoV values, as well as trends with increasing walking speed.

## Discussion

With this analysis, we aimed to determine (1) if we could replicate the statistically significant effects of walking speed on the spatiotemporal parameters and their variability found previously^10^ and (2) if the means and variability of the parameters determined in this way were similar to those reported in a dedicated gait analysis protocol^10^ in a different cohort of people with BVP. Regarding the first aim, in broad alignment with the previous study, we found significant effects of walking speed for all parameters except for step width mean values (which while statistically significant in the previous study, did not show large or clear differences across the walking speeds, as shown in Figure 1). Regarding the second aim, the outcomes appeared to be largely comparable in terms of mean and CoV values, as well as the walking speed relationships. Potential minor exceptions to this could be the double support time mean values and the step width CoV values.

Concerning the few parameters that appeared to slightly differ in Figures 1 and 2, the double support time mean values may be related to two differences in the experimental contexts. The lack of a force plate and therefore the reliance on the markers to determine foot touch down and toe-off reduce the temporal accuracy and consequently the accuracy of the double support time values. A few frames difference at 100 Hz of the motion capture system would correspond to the approximate range of differences of between about 0.03 and 0.08 seconds. Another possibility is that the DVA assessment had a similar effect on participants as is often seen in cognitive dual tasks, namely, an increase in double support time,^24,25^ though this is usually related to a decrease in walking speed. As our results across multiple walking speeds show, the slightly higher double support time in the current DVA-derived data was not due to walking speed differences.

While a significant effect of increasing walking speed on step width variability was found in the current analysis, Figure 2 shows that the effect may not be as strong in the current DVA-derived analysis compared to the previous analysis in McCrum et al.^10^ This may be related to the difference in treadmill width. The CAREN, as used in the previous study, includes a dual belt treadmill 1 metre in belt width, whereas the DVA in the current analysis was conducted on a more typical treadmill with 60 cm belt width. While it did not seem to affect the mean step width (Figure 1), the reduced treadmill width may have constrained the possibility for participants to vary their step width during the trials.

The current study provides a conceptual replication^26^ of earlier findings, indicating the potential for concurrent DVA and gait analysis in people with BVP. However, it should be kept in mind that the replication is at group level and is not synonymous with repeatability or reliability at the individual level. Before considering gait analysis during the DVA assessment in clinical settings, investigation of the test-retest reliability would be advisable. A healthy reference dataset would also allow further investigation into the diagnostic potential of this approach. The differences found between people with BVP and healthy participants in our previous study^10^ were based on the dedicated gait analysis with motion capture on the CAREN system and it is possible that the healthy participants would also show slightly different results when assessed on a regular treadmill while performing a DVA assessment. Sensitivity to change as a result of interventions in BVP is also something not yet extensively investigated in either setup and would be important for clinicians when using such a combined approach. Clinical feasibility of combined DVA and gait assessment would be further increased by using more clinically feasible and affordable methods (e.g., wearable sensors or markerless motion capture systems) to assess gait parameters rather than camera-based 3D motion capture systems. While these are gradually being used more to assess physical activity in BVP,^9,27^ their accuracy and reliability for assessing gait parameters compared to 3D motion capture has not been assessed in people with BVP. In other populations, this has been extensively researched^28–30^ and two important considerations have emerged: achieving accurate detection of gait events (i.e., heel strike and toe-off) is critical but challenging^31,32^; and while assessment of temporal parameters (e.g., step time) can be accurate,^28,33^ assessment of spatial parameters (i.e., step length) is challenging.^33^ Whatever system is used in the future, optimal setup and data quality checks should be put in place.

## Statements and declarations
